# A randomized placebo-controlled phase II study of clarithromycin or placebo combined with VCD induction therapy prior to high-dose melphalan with stem cell support in patients with newly diagnosed multiple myeloma

**DOI:** 10.1186/s40164-018-0110-0

**Published:** 2018-08-13

**Authors:** Henrik Gregersen, Trung Do, Ida Bruun Kristensen, Ulf Christian Frølund, Niels Frost Andersen, Lene Kongsgaard Nielsen, Christen Lykkegaard Andersen, Tobias Wirenfeldt Klausen, Annette Juul Vangsted, Niels Abildgaard

**Affiliations:** 1 Department of Hematology, Aalborg University Hospital, Mølleparkvej 4, 9000 Aalborg, Denmark; 2 Department of Hematology, Herlev Hospital, 2730 Herlev, Denmark; 3 Department of Hematology, Vejle Hospital, 7100 Vejle, Denmark; 4 Department of Hematology, Zealand University Hospital, 4000 Roskilde, Denmark; 5 Department of Hematology, Aarhus University Hospital, 8000 Aarhus, Denmark; 6 Quality of Life Research Center OUH, Odense University Hospital, 5000 Odense C, Denmark; 7 Department of Hematology, Rigshospitalet, 2100 Copenhagen, Denmark; 8 Department of Hematology, Odense University Hospital, 5000 Odense C, Denmark

**Keywords:** Multiple myeloma, Clarithromycin, Bortezomib, Adverse drug event, Induction chemotherapy, Double blind study

## Abstract

**Background:**

The objective of this randomized placebo-controlled study was to investigate the efficacy and safety of clarithromycin in combination with bortezomib–cyclophosphamide–dexamethasone (VCD) in patients with newly diagnosed multiple myeloma eligible for high-dose therapy.

**Methods:**

Patients were randomized to receive tablet clarithromycin 500 mg or matching placebo tablet twice daily during the first 3 cycles of VCD induction therapy. Primary endpoint was to compare the rate of very good partial response (VGPR) or better response after three cycles of VCD combined with clarithromycin or placebo.

**Results:**

The study was prematurely stopped for safety reasons after the inclusion of 58 patients (36% of the planned study population). The patients were randomly assigned to clarithromycin (n = 27) or placebo (n = 31). VGPR or better response after the VCD induction therapy was obtained in 12 patients (44.4%, 95% CI 25.5–64.7) and in 16 patients (51.6%, 33.1–69.8) (p = 0.59) in the clarithromycin group and the placebo group, respectively. Seven patients (25.9%) in the clarithromycin group developed severe gastrointestinal complications (≥ grade 3) comprising pain, neutropenic enterocolitis, paralytic ileus or peptic ulcer. These complications occurred in only one patient in the placebo group. Septicemia with Gram negative bacteria was observed in 5 patients in the clarithromycin group in contrast to one case of pneumococcal septicemia in the placebo group. Patient-reported QoL were negatively affected in the clarithromycin group compared to the placebo group.

**Conclusion:**

The study was prematurely stopped due to serious adverse events, in particular serious gastrointestinal complications and septicemia. The response data do not suggest any effect of clarithromycin when added to the VCD regimen. The combination of clarithromycin and bortezomib containing regimens is toxic and do not seem to offer extra anti-myeloma efficacy.

*Trial registration* EudraCT (no. 2014-002187-32, registered 7 October 2014, https://www.clinicaltrialsregister.eu/ctr-search/trial/2014-002187-32/DK) and ClinicalTrials.gov (no NCT02573935, retrospectively registered 12 October 2015, https://www.clinicaltrials.gov/ct2/show/NCT02573935?term=Gregersen&cntry=DK&rank=9)

## Background

Clarithromycin has been proposed as a potentially good candidate for addition to multiple myeloma therapy in pursuit of synergistic effects [1]. This concept is based on the favorable toxicity profile of clarithromycin when used in the treatment of infections, the very low cost and response data from combination with immunomodulatory drugs (IMiDs) in phase II trials and in one case-matched study [2–5]. In a study by Niesvizky et al. the combination of clarithromycin, 500 mg twice daily with lenalidomide and dexamethasone led to partial response or better in 90% of treatment-naive patients with symptomatic multiple myeloma [4]. Gay et al. conducted a case-matched analysis based on the Niesvizky study and compared 72 patients treated with clarithromycin, lenalidomide and dexamethasone with an equal number of patients seen at the Mayo Clinic only treated with lenalidomide and dexamethasone [5]. The study indicated a very favorable effect of clarithromycin with a higher frequency of complete response (CR), and very good partial response (VGPR) or better in the clarithromycin group. In addition, time-to-progression and progression-free survival were longer in the clarithromycin group. However, there are so far no data from randomized controlled studies to support an effect of clarithromycin in multiple myeloma.

Cellular studies have shown that clarithromycin attenuates autophagy in myeloma cells at clinically relevant concentrations (6–50 μg/mL) [6]. The combination of clarithromycin and bortezomib results in increased cytotoxicity compared to bortezomib alone in myeloma cell lines [7]. A possible mechanism underlying this synergistic effect might be simultaneous inhibition of the ubiquitin–proteasome system by bortezomib and the autophagy-lysosome system by clarithromycin resulting in over-loading endoplasmic reticulum-stress in myeloma cells [7]. However, it is unknown whether this observation could be translated into clinical efficacy in treatment of multiple myeloma patients.

The Danish Myeloma Study Group (DMSG) therefore initiated a randomized, double-blind, placebo-controlled phase 2 study to evaluate the efficacy and safety of adding clarithromycin to the bortezomib-containing triplet induction regimen bortezomib–cyclophosphamide–dexamethasone (VCD) in multiple myeloma patients eligible for high-dose melphalan with stem cell support (HDT).

## Methods

### Trial design

This randomized, double-blind, placebo-controlled phase II study was designed and conducted by the Danish Myeloma Study Group (DMSG). It included multiple myeloma patients from six Danish sites and was planned to include a total of 160 patients. The study was approved Danish Health and Medicines Authority (No. 2014061645) and Danish Data Protection Agency (No. 2008-58-0028). EudraCT and ClinicalTrials.gov Numbers are 2014-002187-32 and NCT02573935, respectively. Independent monitors from the Danish Good Clinical Practice units in Copenhagen, Aarhus and Odense carried out the monitoring.

### Patients

The study included patients with newly diagnosed multiple myeloma with treatment demanding disease according to the International Myeloma Working Group (IMWG) criteria [8]. Only patients eligible for high-dose melphalan with stem cell support were included. The key exclusion criteria were any given anti-myeloma treatment prior to inclusion, except radiotherapy, bisphosphonates/denosumab or corticosteroids for symptom control, prolonged QT corrected (QTc) interval (> 500 ms on screening ECG), uncontrolled or severe cardiovascular disease, severe renal dysfunction (estimated creatinine clearance < 10 mL/min) and concurrent treatment with certain potentially interacting medications, e.g. fluconazole, verapamil and simvastatin.

### Trial treatment

Patients were randomized (1:1 ratio) to receive oral clarithromycin 500 mg or a matching placebo tablet twice daily during the first 3 cycles of VCD induction therapy. The randomization was stratified according to International Staging System stage (1, 2 or 3). The VCD consisted of 21-day cycles of subcutaneous bortezomib 1.3 mg/sqm days 1, 4, 8, 11, intravenous cyclophosphamide 500 mg/sqm on days 1 and 8, and oral dexamethasone 40 mg days 1, 2, 4, 5, 8, 9, 11, 12. The number of VCD series was changed from three to four in the national Danish guidelines for treatment of multiple myeloma during conduct of the study. Consequently, after a protocol amendment the induction therapy was changed to four series of VCD but the treatment duration of clarithromycin or placebo was unchanged. After the induction therapy the patients proceeded to cyclophosphamide priming (2000 mg/sqm), peripheral blood stem cell harvest by leukapheresis and high-dose melphalan (200 mg/sqm) with stem cell support.

### End-points and assessments

The primary end-point of the study was to compare the rate of very good partial response or better response (≥ VGPR) after three courses of VCD combined with clarithromycin or placebo. The response was assessed according to the International Myeloma Working Group criteria for response in multiple myeloma [9]. An important secondary end-point was to compare the rate of ≥ VGPR 2 months after high-dose melphalan with stem cell support. Other secondary end-points included the frequency of infections and the number of stem cells harvested in patients in the two treatment groups. Patient-reported quality of life (QoL) and neurotoxicity were secondary end-points, and assessed at inclusion and after 2 and 6 months. Two European Organisation for Research and Treatment of Cancer QoL (EORTC) questionnaires were used; EORTC QLQ-C30 and the Multiple Myeloma module EORTC QLQ-MY20 [10, 11]. Neurotoxicity was assessed by the Functional Assessment of Cancer Therapy/Gynecologic Oncology Group-Neurotoxicity (FACT/GOG-Ntx) subscale questionnaire [12]. Analyses were by intention-to-treat.

### Safety assessment

Adverse events were graded according to National Cancer Institute Common Toxicity Criteria (NCI CTC) Version 4.0.

### Unblinding

Unblinding of study drug status of the individual patient was performed after final study evaluation 2 months after HDT provided that all CRFs had been completed and approved by the study office. This approach was used to allow for participation in other myeloma studies or the use of tandem transplantation in selected patients.

### Statistical analysis

Comparisons of binary variables were conducted by Fisher’s exact test, mid-P approach as appropriate. Differences in primary endpoints between treatment arms were presented using absolute risk difference with 95% confidence intervals (95% CI). Continuous variables were presented using medians with range or interquartile range. Continuous and ordinal variables were compared using Mann–Whitney test except for patient related outcomes. For analyzing time to exclusion the Kaplan–Meier method was used and differences between treatment arms were compared using the Log-rank test. Differences of patient related outcomes between groups were adjusted for baseline measures and analyzed using a constrained longitudinal data analysis (cLDA) estimated by a mixed model using unstructured covariance [13]. All confidence intervals are 95% and all confidence intervals and p-values are two-sided. Data analyses were performed using R version 3.3.3 (R Foundation for Statistical Computing, Vienna, Austria) except for mixed models which were performed using SAS version 9.4 (SAS institute, Cary, SC, USA).

## Results

The study was prematurely stopped by the study safety board for safety reasons after inclusion of 58 patients (36% of the planned study population). The median age of included patients was 63 years (interquartile range 55–66 years). Twenty-seven patients were assigned to clarithromycin treatment and 31 to placebo. The clinical characteristics at baseline are described in Table 1.

**Table 1:** Baseline characteristics of patients

		Clarithromycin group (N = 27)	Placebo group (N = 31)
Variable			
Age (years)	Median (IQR)	63 (55.0–66.5)	63 (55.5–66.0)
Male sex	No. (%)	20 (74.1%)	20 (64.5%)
Type of myeloma	No. (%)		
IgA		3 (11.1%)	9 (29.0%)
IgG		20 (74.1%)	17 (54.8%)
Light chain		4 (14.8%)	5 (16.1%)
International staging system	No. (%)		
I		7 (%) (25.9%)	9 (29.0%)
II		10 (%) (37.0%)	17 (54.8%)
III		9 (33.1%)	4 (12.9%)
Missing		1 (3.8%)	1 (3.2%)
Cytogenetic features	No. (%)		
Standard risk		18 (66.7%)	18 (58.1%)
High risk^a^		6 (22.2%)	8 (25.8%)
Data not available		3 (11.1%)	5 (16.1%)
WHO performance status	No. (%)		
0		14 (51.9%)	17 (54.8%)
≥ 1		13 (48.1%)	14 (45.2%)
Serum creatinine ≥ 130 µmol/L	No. (%)	5 (18.5%)	2 (6.5%)
Serum LDH ≥ 260 U/L	No. (%)	1 (3.7%)	4 (12.9%)
Serum C-reactive protein ≥ 8 mg/L	No. (%)	9 (33.3%)	9 (29.0%)

*IQR* interquartile range, *LDH* lactate dehydrogenase

^a^t(4;14), t(14;16), t(14;20) or del(17p)

### Efficacy

The primary end-point VGPR or better response after the VCD induction therapy was obtained in 12 patients (44.4%, 95% CI 25.5–64.7%) and in 16 patients (51.6%, 33.1–69.8%) (p = 0.59) in the clarithromycin group and the placebo group, respectively. There were no differences between the two groups for any of the secondary endpoints VGPR or better response 2 months after high-dose melphalan with stem cell support, yield of harvested stem cells and number of infections (Table 2).

**Table 2:** Clinical outcomes

		Clarithromycin group (N = 27)	Placebo group (N = 31)	P_clarithromycin_ − P_control_
Clinical outcomes				
VGPR or better response after VCD induction	No. (%, 95% CI)	12 (44.4%, 25.5–64.7%)	16 (51.6%, 33.1–69.8%)^a^	− 7.2% (− 30.7–17.6%)^c^
VGPR or better response after HDT	No. (%, 95% CI)	16 (59.3%, 38.8–77.6%)	23 (74.2%, 55.4–88.1%)^a^	− 14.9% (− 37.1–8.9%)^c^
Leukapheresis	10^6^/kg (range)	8 (2–20)	8.5 (2–18)^b^	–
Any infection	No. (%, 95% CI)	16 (59.3%, 38.8–77.6%)	18 (58.1%, 39.1–75.5%)^a^	1.2% (− 23.0–24.9%)^c^

*VGPR* very good partial response, *HDT* high-dose melphalan with hematopoietic stem cell support

^a^Not significant (Fisher’s exact test)

^b^Not significant (Mann–Whitney test)

^c^P_clarithromycin_ − P_control_ For response negative numbers indicate lower response in clarithromycin group. For infection positive number indicates higher risk of infection in clarithromycin group

### Safety

Most patients in the clarithromycin group and the placebo group had at least one adverse event during the VCD induction therapy (96.3% and 90.3%, respectively) (Table 3). The most common adverse events of any grade are summarized in Table 4. Frequent adverse events in the clarithromycin group were thrombocytopenia, septicemia, oral candidiasis, peripheral sensory neuropathy (p = 0.03), dizziness, peripheral edema, hypotension and various psychiatric symptoms. By contrast, the occurrence of respiratory tract infection was low in the clarithromycin group.

**Table 3:** Overall safety profile and drug doses in the clarithromycin group and placebo group

	Clarithromycin group, No = 27	Placebo group, No = 31
120/180 days follow-up—%	77.8% (63.6%; 95.2%)	90.3% (80.5%; 100%)
Treatment cycles—no. (%)		
1	1 (3.7%)	2 (6.4%)
2	3 (11.1%)	1 (3.2%)
3	16 (59.3%)	19 (61.2%)
4	7 (25.9%)	9 (29.0%)
Any adverse event—no. (%)	26 (96.3%)	28 (90.3%)
Any ≥ 3 adverse event—no. (%)	16 (59.3%)	12 (38.7%)
Any serious adverse event—no. (%)	16 (59.3%)	10 (32.3%)
Adverse event resulting in dose reduction of study drug—no. (%)	9 (33.3%)	3 (9.7%)^a^
Adverse event resulting in dose reduction of bortezomib—no. (%)	9 (33.3%)	4 (12.9%)^b^
Adverse event resulting in dose reduction of dexamethasone—no. (%)	9 (33.3%)	3 (9.7%)^a^
Adverse event resulting in dose reduction of cyclophosphamide—no. (%)	4 (14.8%)	1 (3.2%)^b^

^a^p = 0.049 (Fisher’s exact test)

^b^Not significant (Fisher’s exact test)

**Table 4:** Most common adverse events in the clarithromycin and placebo group

	Clarithromycin group, No = 27		Placebo group, No = 31	
	Any grade	Grade 3 or 4	Any grade	Grade 3 or 4
Hematologic events				
Thrombocytopenia	11 (40.7%)	2 (7.4%)	7 (22.6%)	0
Anemia	12 (44.4%)	2 (7.4%)	11 (35.5%)	0
Neutropenia	11 (40.7%)	1 (3.7%)	13 (41.9%)	0
Gastrointestinal events				
Typhlitis and perforation of colon	2 (7.4%)	2 (7.4%)	1 (3.2%)	1 (3.2%)
Paralytic ileus	2 (7.4%)	2 (7.4%)	0	0
Constipation	6 (22.2%)	1 (3.7%)	5 (16.1%)	0
Diarrhea	4 (14.8%)	3 (11.1%)	5 (16.1%)	1 (3.2%)
Dyspepsia	3 (11.1%)	0	1 (3.2%)	0
Nausea	5 (18.5%)	0	6 (19.4%)	0
Infections				
Respiratory tract infection	5 (18.5%)	2 (7.4%)	8 (25.8%)	5 (16.1%)
Urinary tract infection	3 (11.1%)	1 (3.7%)	5 (16.1%)	1 (3.2%)
Septicemia	5 (18.5%)	5 (18.5%)	1 (3.2%)	1 (3.2%)
Other infections	6 (22.2%)	4 (14.8%)	9 (29.0%)	5 (16.1)
Oral candidiasis	7 (25.9%)	1 (3.7%)	1 (3.2%)	0
Nervous system disorders				
Peripheral sensory neuropathy	15 (55.6%)	0	8 (25.8%)	0
Dizziness	4 (14.8%)	1 (3.7%)	3 (9.7%)	0
Other conditions				
Peripheral edema	11 (40.7%)	1 (3.7%)	7 (22.6%)	1 (3.2%)
Hypotension	4 (14.8%)	2 (7.4%)	1 (3.2%)	0
Fatigue	4 (14.8%)	1 (3.7%)	3 (9.7%)	0
Rash	2 (7.4%)	0	4 (12.9%)	0
Insomnia	2 (7.4%)	0	6 (19.4%)	0
Weight loss	3 (11.1%)	0	1 (3.2%)	0
Mucositis	2 (7.4%)	0	4 (12.9%)	0
Psychiatric symptoms^a^	5 (18.5%)	1 (3.7%)	2 (6.5%)	1 (3.2%)

The table included adverse event of any grade that occurred in more than 10% in any of the treatment groups or adverse events grade 3 or more that occurred in more than 5% in any of the treatment groups

^a^The group psychiatric symptoms encompass anxiety, agitation, depression and psychosis

A total of 26 serious adverse events (SAEs) were reported in 16 (59.3%) patients in the clarithromycin group and 16 SAEs in 10 (32.3%) patients in the placebo group. A large fraction of the SAEs was constituted by gastrointestinal complaints with associated serious complications which were seen in 7 (25.9%) patients in the clarithromycin group and in 1 patient (3.2%) in the placebo group. A common complication in patients with gastrointestinal complaints in the clarithromycin group was septicemia which was detected in 5 cases and constituted well-known gastrointestinal bacteria. By contrast, the only case of septicemia detected in the placebo group was pneumococcal sepsis associated with pneumonia. There was one death in the clarithromycin group (duodenal ulcer) and one death in the control group (perforated diverticulitis). As a consequence of the imbalance in gastrointestinal symptoms and septicemia the study safety board decided to terminate inclusion of new patients and administration of the study drug ceased on 16 September 2016. The VCD treatment and follow-up in the study continued.

### Treatment

The fraction of patients who either had a reduced dose of study drug or discontinued study drug due to adverse events was higher in the clarithromycin group compared with the placebo group (33.3% and 9.7%, respectively). In addition, there was a trend towards more frequent reduction of VCD drug doses in the clarithromycin group compared with the placebo group, e.g. for bortezomib 33.3% and 12.9%, respectively (Table 3).

### Patient-reported outcomes

**Fig. 1:**
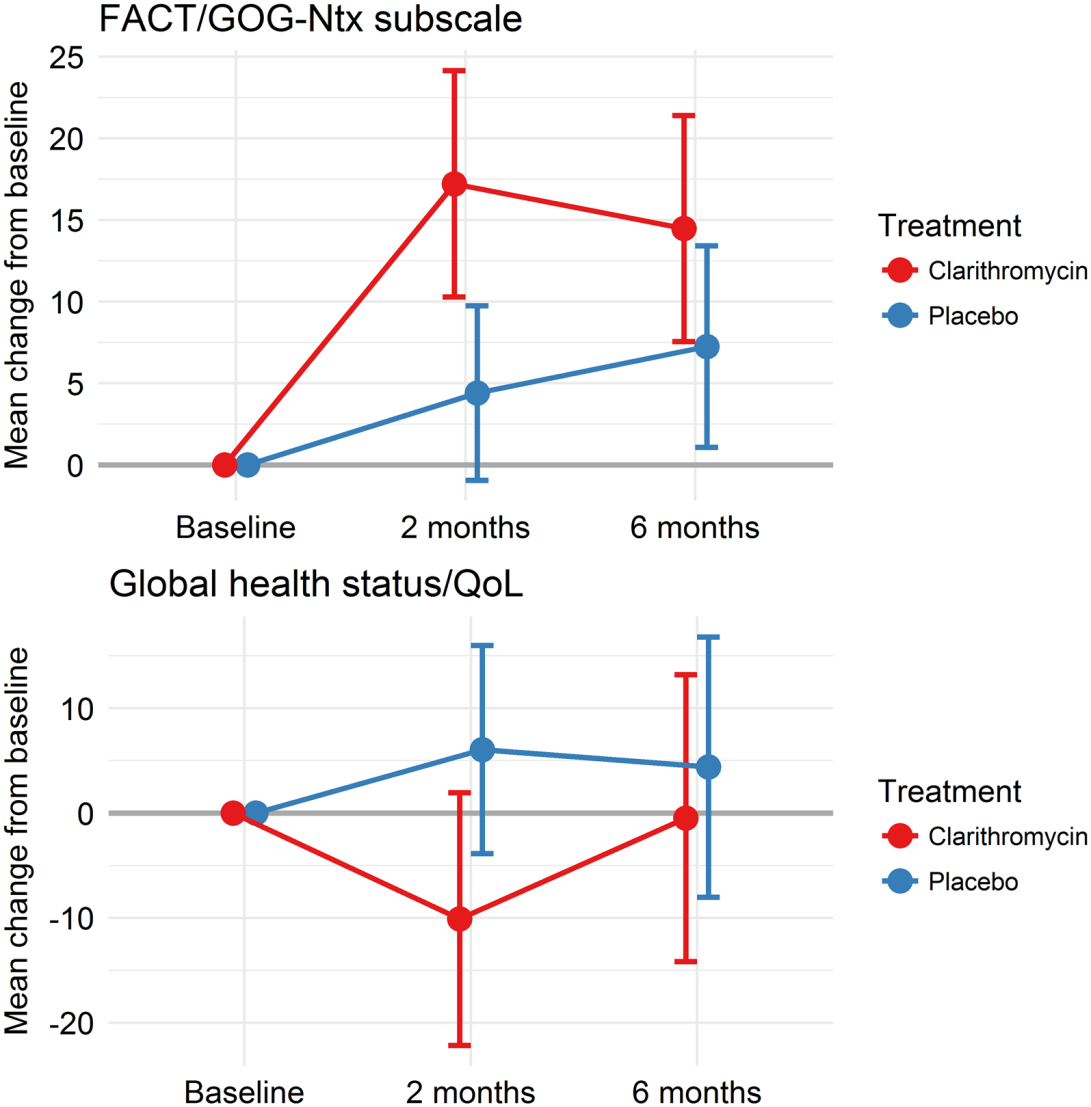
Patient reported neurotoxicity and global quality of life in the clarithromycin and the placebo group. In the FACT/GOG-Ntx score positive values indicate worsening of neurotoxicity and negative values indicate improvement of neurotoxicity. In the Global Quality of Life Scale (GQOL) positive values indicate improvement in quality of life and negative values indicate worsening of quality of life

Patients in the clarithromycin group reported more and clinical relevant neurotoxicity on the FACT/GOG-Ntx subscale and clinical relevant reduced global Qol on the EORTC QLQ C30 than patients in the placebo group after three VCD series. Two month after HDT there was still a mean score difference between the two treatment groups, but the differences were not clinical relevant anymore (Fig. 1).

## Discussion

To our knowledge, this study is the first randomized placebo-controlled trial examining the effect of adding clarithromycin to conventional myeloma therapy. The study was prematurely stopped for safety reasons, which affect the interpretation of the results, but our data do not suggest any anti-myeloma effect of clarithromycin when added to the VCD regimen.

The case-matched retrospective analysis by Gay et al. found a very pronounced effect of adding clarithromycin to treatment with lenalidomide and dexamethasone, e.g. tripling of the CR rate [5]. The lack of efficacy of clarithromycin in combination with CVD in our study does not rule out a significant and clinical meaningful effect of clarithromycin in combination with other myeloma drugs, e.g. the immunomodulatory agents (IMiDs). The potential effect of the combination of clarithromycin and lenalidomide is to our knowledge currently being assessed in two active recruiting randomized controlled trials.

At the planning of our study a major concern was potential serious cardiac side effects since clarithromycin may increase the QT interval, and the drug has been associated with risk of cardiovascular events and increased mortality in patients with stable coronary heart disease and in patients without heart disease [14, 15]. The exclusion criteria in our study were therefore strict in regard to previous cardiovascular disease and concomitant use of drugs that might increase the QT interval. Maybe due to this, we did not encounter any serious cardiovascular morbidity during conduct of the study.

Surprisingly, we observed an increased occurrence of several types of adverse events in patients who received clarithromycin in combination with VCD and several mechanisms may underlie this finding. First, bortezomib is metabolized by the cytochrome P450 (CYP) enzymes, and in particular the CYP3A4 is the major contributor to bortezomib metabolism [16]. Secondly, clarithromycin inhibits CYP3A4 and probably the observed adverse events in our study were merely an effect of increased biological bortezomib exposure due to reduced metabolism [17]. Peripheral neuropathy is a common adverse event during treatment with bortezomib and we found a clear difference between the two treatment groups in our study [18]. In accordance with this observation an increased occurrence of peripheral neuropathy and also thrombocytopenia has been observed in patients who received concomitantly bortezomib and itraconazole, another potent CYP3A4 inhibitor [19]. Possibly, this mechanism might explain some of the observed gastrointestinal symptoms, e.g. increased occurrence of constipation and paralytic ileus in our study. In addition, other factors might have contributed, e.g. clarithromycin increases the pharmacologic effect of steroids and cases of pseudomembranous colitis have been observed in patients treated with clarithromycin as part of eradication therapy for Helicobacter pylori infection [4, 20–22]. In accordance with our findings an increased rate of grade 3–4 adverse events was also observed in the clarithromycin group in the case-matched study by Gay et al. [5], and noteworthy three cases of perforated colon occurred in the clarithromycin group in contrast to none in the control group. However, the occurrence of septicemia was the same in the groups in the study by Gay et al. which is in contrast to our results [5]. Although most of the measures of safety in our study did not reach statistical significance our data consistently suggest an unfavorable pattern of clarithromycin combined with VCD on the number and degree of adverse events, on feasibility of the regimen and on the two patient-reported outcomes quality of life and neurotoxicity. Such a pattern is not acceptable in an era of novel potent anti-myeloma drugs with favorable safety profiles where in particular the monoclonal antibodies daratumumab and elotuzumab are likely to constitute important elements of bortezomib-containing regimens [23, 24].

In conclusion, although we were only able to analyze response data in 58 included patients, our data do not indicate any additional effect of clarithromycin when added to the VCD regimen, and due to treatment toxicity our trial does not encourage further clinical studies on the combination of clarithromycin and bortezomib. In patients treated with combined clarithromycin and VCD we observed an increased frequency of serious adverse events, in particular serious gastrointestinal complications and septicemia. This emphasizes the need for controlled studies on the efficacy of clarithromycin, both in assessment of potential anti-myeloma effects as well as for assessment of safety measures.
